# Fungal spore swelling and germination are restricted by the macrophage phagolysosome

**DOI:** 10.1016/j.funbio.2023.08.002

**Published:** 2023-09

**Authors:** María Fernanda Alonso, Judith M. Bain, Lars P. Erwig, Alistair J.P. Brown, Neil A.R. Gow

**Affiliations:** a Aberdeen Fungal Group, School of Medicine, Medical Sciences & Nutrition, Institute of Medical Sciences, University of Aberdeen, Foresterhill, Aberdeen, AB25 2ZD, UK; b Medical Research Council Centre for Medical Mycology, University of Exeter, Geoffrey Pope Building, Stocker Road, Exeter, EX4 4QD, UK

**Keywords:** *Aspergillus*, Fungal spore, Macrophage, Medical mycology, *Mucor*

## Abstract

Many species of medically important fungi are prolific in the formation of asexual spores. Spores undergo a process of active swelling and cell wall remodelling before a germ tube is formed and filamentous growth ensues. Highly elongated germ tubes are known to be difficult to phagocytose and pose particular challenges for immune phagocytes. However, the significance of the earliest stages of spore germination during immune cell interactions has not been investigated and yet this is likely to be important for defence against sporogenous fungal pathogens. We show here that macrophages restrict the early phases of the spore germination process of *Aspergillus fumigatus* and *Mucor circinelloides* including the initial phase of spore swelling, spore germination and early polarised growth. Macrophages are therefore adept at retarding germination as well as subsequent vegetative growth which is likely to be critical for immune surveillance and protection against sporulating fungi.

## 1 Introduction

The majority of the estimated 2-5 million fungal species form airborne asexual spores (Blackwell, 2011; Hawksworth and Lücking, 2017), which inevitably will be sucked into the lungs of humans and other mammals. For the medically relevant fungus *Aspergillus fumigatus* it is estimated, for example, that several hundreds or thousands of spores impact on the respiratory mucosa every day (Latgé, 1999). Therefore, it is vital that the innate immune system in the lung efficiently recognises, clears and kills potential fungal infections at this site. Macrophages can only engulf part of a long hypha, and in so doing they generate phagosomes that may not be completely sealed, but are still partially effective in containing extended hyphal growth (Maxson et al., 2018). Macrophages can also actively fold hyphae to facilitate their complete engulfment (Bain et al., 2021). However, there are advantages to being able to recognise and restrict fungal growth at the earliest phases of lung colonisation, before hyphae have formed. Yet little is known about the ability of phagocytes to restrict fungal spore germination, and it is this aspect of the host–fungus interaction that is addressed in this study. We examine this using two examples of genetically distinct and evolutionarily divergent sporulating fungi that are both major human pathogens - *Aspergillus fumigatus* and *Mucor circinelloides* (World Health Organization, 2022). Though evolutionary distant from one another, these opportunistic moulds share some common pathophysiological features. Both fungi can cause disease in susceptible individuals when inhaled airborne fungal spores initiate infection in the respiratory tract, and neither can be contained by resident or recruited phagocytes. Such impaired innate immune responses allow spores to germinate into tissue-penetrating hyphae. Fungal hyphae can invade the sinuses or the lung parenchyma, reach blood vessels and disseminate to more distant tissues (e.g. brain, liver) (Köhler et al., 2014). These invasive infections are often refractory to treatment and hence are life-threatening.

Before they germinate, most fungal spores swell, and their walls become progressively thinner (Gull and Trinci, 1971). Given the importance of mycelial growth in the pathobiology of invasive aspergillosis and mucormycosis, the prevention of spore germination (spore swelling and germ tube emergence) and early killing of hyphal cells are essential features of an effective immune response. In immunocompetent hosts, distinct phagocyte populations fulfil these roles and prevent disease onset. Macrophages are crucial for the inhibition of spore swelling (*A. fumigatus* and *M. circinelloides*) (Philippe et al., 2003; Waldorf et al., 1984a; 1984b) and killing of fungal spores (*A. fumigatus*) (Gilbert et al., 2014; Levitz et al., 1986; Waldorf et al., 1984a; Xu and Shinohara, 2017). Neutrophils are essential for killing germ tubes and hyphal cells via oxidative and non-oxidative mechanisms (Levitz et al., 1986; Waldorf and Diamond, 1985). Several groups have shown this division of labour between neutrophils and macrophages *in vitro* and *in vivo* (Rudkin et al., 2013; Knox et al., 2014; Voelz et al., 2015).

Conidia and other spores have components of the wall surface that are not recognised by immune receptors or that are shielded by superficial masking components of the wall. For example, the hydrophobin RodA covers conidial surfaces and is immunologically inert thereby preventing access to some of the sub-superficial spore wall components (Aimanianda et al., 2009; Gow and Lenardon, 2023). Spore dihydroxynaphthalene (DHN) melanin can be detected my MelLec and is an important aspect of *Aspergillus* immune recognition that results in metabolic rewiring of macrophages (Gonçalves et al., 2020; Stappers et al., 2018). Melanin is a substrate for surfactant protein on the surface of *Aspergillus* conidia, and this contributes to opsonisation and efficient conidium uptake (Wong et al., 2018). Melanin is also an important component of the conidia of Mucorales species (Nicolás et al., 2020) and is retained during swelling of *Rhizopus* conidia resulting in phagosomal maturation arrest and inhibition of phagocytosis. The conidium of *Rhizopus* can retain melanin that can induce persistence in alveolar macrophages by retarding phagosome maturation and inhibition of phagocytosis (Andrianaki et al., 2018). Some Mucorales species can survive in a dormant state inside macrophages and some *Rhizopus* species germinate and escape from immune phagocytes (Nicolás et al., 2020). During spore swelling new sub-superficial components of the spore may become exposed as the surface layer stretches or cracks. However, in a study using J774A.A monocyte-macrophages cell lines, swollen spores were paradoxically shown to be less efficiently phagocytosed than dormant spores (Itabangi et al., 2022), and in another study *Rhizopus* spores were shown phagocytosed as efficiently as swollen spores (Voelz et al., 2015).

Inside the host, in the presence of water, oxygen and appropriate carbon, phosphate and nitrogen sources, inhaled spores can initiate the process of germination (Ekundayo and Carlile, 1964; Osherov, 2009). For most fungal spores, including the asexual spores of *A. fumigatus* and *M. circinelloides,* this is a two-stage process. First, resting spores grow isotropically by taking up water and synthesising new cell wall material. This phase is known as “swelling” (Bartnicki-García and Lippman, 1977; Osherov, 2009). It is followed by a phase of polarized growth and emergence of a germ tube. The germ tubes continue to grow by polarised apical extension to become hyphae (Bartnicki-García and Lippman, 1977; Momany and Hernández-Rodríguez, 2009). Unlike the spherical conidia of *A. fumigatus*, sporangiospores of *M. circinelloides* are ellipsoidal. Previous studies have described an initial stage of swelling for spores of *Mucor* spp. where the spore changes shape from ellipsoidal to spherical, initially growing faster in the minor axis before initiating isodiametric growth (Bartnicki-García and Lippman, 1977; Cano and Ruiz-Herrera, 1988). Germination in *A. fumigatus* is a synchronous process (Momany and Taylor, 2000). This is not the case for *M. circinelloides*. Spore populations of various clinically relevant strains exhibit size heterogeneity, with larger cells showing shorter isotropic growth phases than smaller cells (Li et al., 2011). The faster emergence of germ tubes in larger cells correlates with increased virulence (Li et al., 2011).

The present study focused on the inhibition of spore swelling by macrophages. Previous research has compared the capacity of different types of murine or human macrophages (e.g. bronchoalveolar, peritoneal) to control spore growth under healthy or predisposing conditions (e.g. immunosuppressive corticosteroid treatment, diabetic ketoacidosis) (Philippe et al., 2003; Waldorf et al., 1984a). These studies used fixed endpoints and measured germ tube emergence from internalized spores as an indicator of macrophage capacity to control infection. By focusing on the final outcome of germination, these approaches do not provide information about the capacity of macrophages to inhibit the individual phases of this process (swelling and polarized growth). Also, it is unclear whether there is a window of opportunity for macrophages to control germination. To our knowledge macrophage inhibition of sporangiospore swelling has not been addressed for members of the Mucoracea family. For *A. fumigatus*, it has been reported that delayed, but not impaired, conidial swelling occurs inside alveolar macrophages compared to unchallenged conidia (Philippe et al., 2003). Temporal dissection of this process is therefore lacking and, here, live cell video microscopy was used to assess individual germination dynamics of internalized and non-internalized spores. Internalized spores were further classified as early or late internalization events to evaluate the capacity of macrophages to control swelling once the process had been initiated.

## 2 Materials and methods

### 2.1 Fungal strains and growth conditions

*A. fumigatus* NIH 5233 and *M. circinelloides* CBS 277.49 were obtained from glycerol stocks stored at -80 °C and grown on potato dextrose agar (PDA) slants (BD Bioscience) for 4–5 days in the dark at 37 °C and room temperature, respectively. Fresh *A. fumigatus* and *M. circinelloides* spores were collected in PBS containing 0.1% Tween-20 and filtered through a 40 μm cell strainer to remove hyphal fragments. Spores were washed twice in PBS (6000 rpm, 3 min) and counted with a haemocytometer.

### 2.2 Thioglycollate-elicited peritoneal mouse macrophages

C57BL/6 female mice were used as a source of peritoneal macrophages and were obtained from specific pathogen-free facilities at the University of Aberdeen and used at ∼10–14 weeks of age. Thioglycollate-elicited peritoneal macrophages were obtained from sacrificed mice 3–4 days after an intraperitoneal injection of 1 ml 3% Brewer’s thioglycollate broth (BD Bioscience). Cells were harvested by flushing the peritoneal cavity with 10 mL ice-cold sterile 5 mM EDTA in phosphate-buffered saline (PBS) and then washed 2 times (400 g, 10 min) with RPMI 1640 Glutamax (Life Technologies) supplemented with 10% (v/v) heat-inactivated foetal calf serum (Sigma), 200 U/mL penicillin/streptomycin (Sigma) and 10 mM HEPES (Life Technologies). For phagocytosis assays, 1.5x10^5^ cells/well were seeded onto 8-well μ-slides (ibiTreat surface, ibidi). Cells were incubated for 20–24 h at 37 °C with 5% CO_2_, after which non-adherent cells were removed by 2 washes with supplemented RPMI 1640 medium.

### 2.3 Live cell imaging

Standard phagocytosis assays were performed as described previously (Lewis et al., 2013). Briefly, spores of *A. fumigatus* and *M. circinelloides* CBS 277.49 were co-incubated with adhered macrophages at a multiplicity of infection (MOI) of 1:1 immediately prior to image acquisition. Interactions were recorded using an UltraVIEW VoX spinning-disk microscope (Nikon) with an environmental control chamber. Images were captured every 2 min for a total of 8 h using a x40 or x 60 objective.

LysoTracker Red (LTR) DND-99 (Invitrogen) at 50 nM was used to pre-stain macrophages that had been grown in supplemented RPMI 1640 medium and incubated for an hour at 37 °C, 5% CO_2_.

### 2.4 Analysis of spore germination dynamics from live cell videos

Image analysis was performed using Volocity 6.3 software (Improvision, Perkin Elmer). For analysis spores were classified in three groups: (i) early internalized (internalization within first 30 min of image acquisition), (ii) late internalized (internalization between 150 and 210 min or 210–270 min of image acquisition for *A. fumigatus* and *M. circinelloides*, respectively) and (iii) non-internalized. Live-cell video microscopy enables internalization of spores to be visualized without the need to stain external spores. However, during these experiments, to further aid visualization of ingested spores, macrophages were stained using the red fluorescent dye LysoTracker red DND-99, which stains acidic compartments (Lewis et al., 2013). Diameters of individual spores were measured every 30 min for 8 h or until germ tube emergence. For *M. circinelloides* major and minor axes of the sporangiospores were measured. Data in main text refers to major axis measurements of *M. circinelloides*. Rates of spore swelling were determined for each individual spore as the slope of the line on the diameter–time graph. For late internalized spores, speed of spore swelling was determined before and after engulfment. Percentages of spore germination were also determined for each group after 8 h of imaging. Data from 3 biologically independent experiments was gathered and, in total, 50 fungal spores per condition were analysed.

### 2.5 Statistical analyses

Datasets were tested using the D'Agostino & Pearson omnibus normality test to determine if they were well modelled by a Gaussian distribution. For non-normally distributed data median values and interquartile ranges were calculated. Statistical significance was assessed by Kruskal–Wallis test followed by Dunn’s multiple comparison test or Mann–Whitney test, as appropriate. A P value of less than 0.05 was considered significant.

### 2.6 Ethics statement

All animal procedures were conducted in accordance with the terms and conditions of the United Kingdom Home Office licence 70/8073 for research on animals and the University of Aberdeen ethical committee. Work involving animals (mice) to generate peritoneal macrophages was carried out in accordance with UK Home Office regulations under an existing Home Office project licence The use of animals in experiments and testing was regulated under the Animals (Scientific Procedures) Act 1986 (ASPA) and European Directive 2010/63/EU.

## 3 Results

### 3.1 Phagosome environment delays swelling of resting *A. fumigatus* and *M. circinelloides* spores

**Fig. 1:**
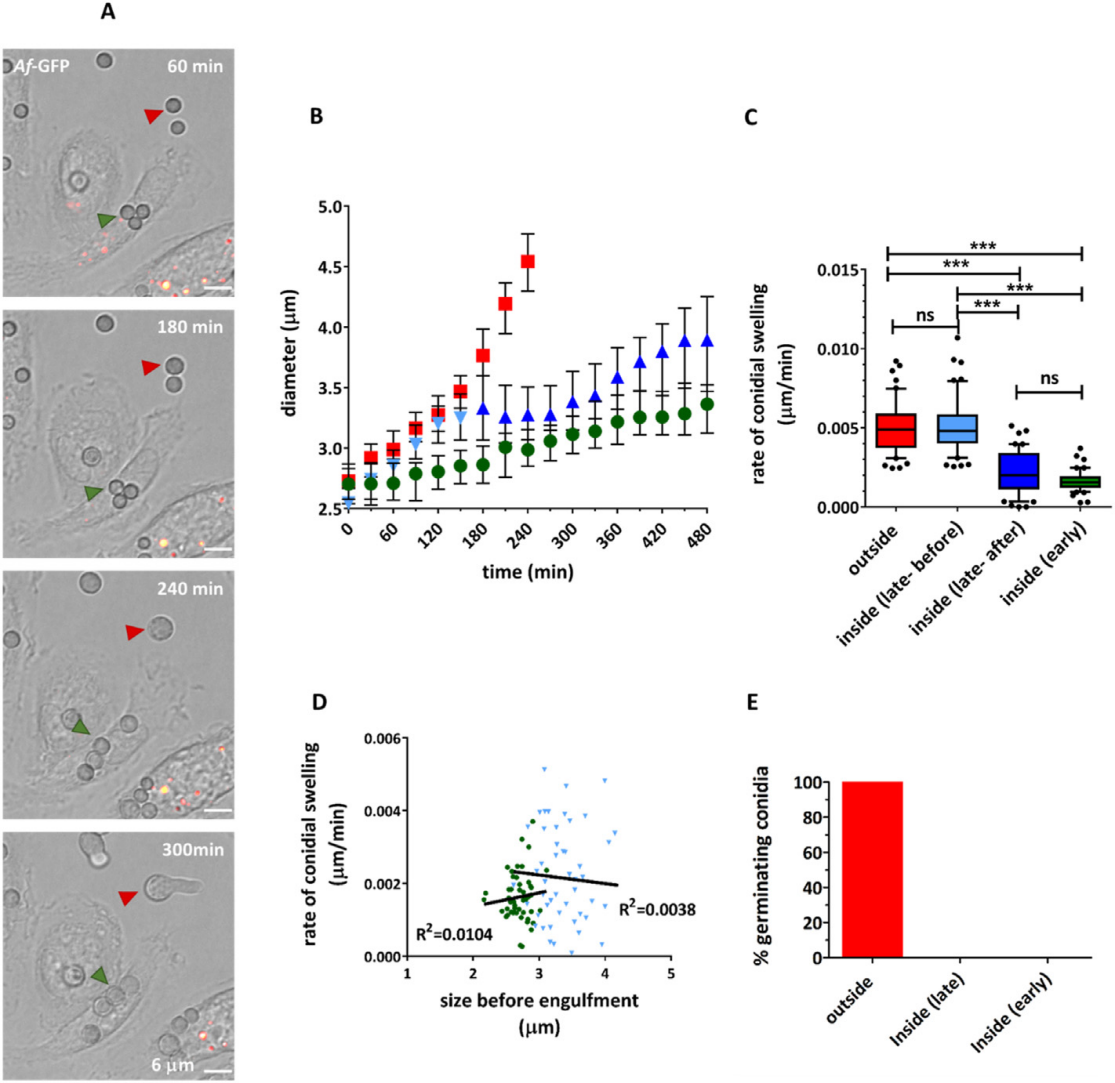
Phagocytic environment delays swelling and inhibits germination of resting and pre-swollen *A. fumigatus* conidia. (A) Representative snapshots from live cell video microscopy of murine thioglycollate-elicited peritoneal macrophages co-incubated with *A. fumigatus* conidia (indicated with arrows). An early internalized conidium (green arrowhead) and a non-internalized conidium (red arrowhead) are indicated (scale bars = 6 μm). Macrophage acidic compartments are stained with LysoTracker Red. B-C) Macrophages can modulate rate of conidial swelling. Growth profiles of non-internalized (), early internalized () and late internalized [before () and after () internalization] *A. fumigatus* conidia (B). Data are represented as median ± IQR of 50 individual conidial measurements acquired over time from 3 biologically independent replicates. Statistical significance was assessed by Kruskal–Wallis test followed by Dunn’s multiple comparison test or Mann–Whitney test, as appropriate. Rate of conidial swelling (slope of diameter–time graph) (C). Data are represented as box and whiskers [IQR (boxes), 10–90 percentile (whiskers), median (horizontal line) and outliers (dots)] of 50 individual conidial measurements acquired from 3 biologically independent replicates. Statistical significance was assessed by Kruskal–Wallis test followed by Dunn’s multiple comparison test. D) No correlation was observed between conidial size prior to engulfment and speed of conidial swelling inside phagocyte. E) Germination of resting and pre-swollen conidia was completely inhibited within the phagocyte. Early internalization = internalized in first 30 min of image acquisition; late internalization = internalized between 150 and 210 min of image acquisition; ∗P≤0.05, ∗∗P ≤0.01, ∗∗∗P ≤0.001, ns = P>0.05.

**Fig. 2:**
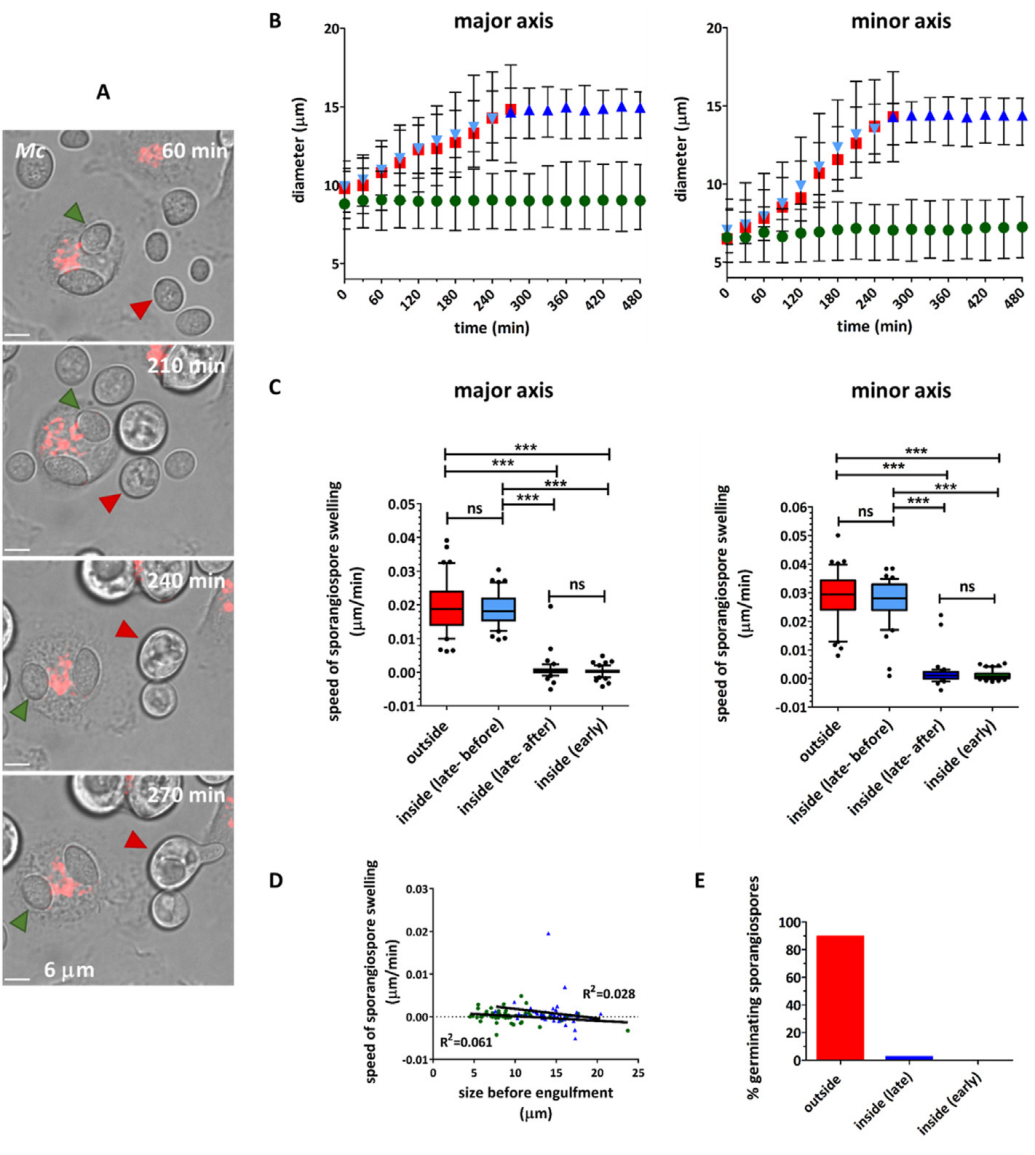
Phagocytic environment inhibits swelling and germination of resting and pre-swollen *M. circinelloides* sporangiospores. (A) Representative snapshots from live cell video microscopy of murine thioglycollate-elicited peritoneal macrophages co-incubated with *M. circinelloides* sporangiospores. Macrophage acidic compartments are stained with LysoTracker Red. An early internalized sporangiospore (green arrowhead) and a non-internalized sporangiospore (red arrowhead) are indicated (scale bars = 6 μm). (B-C) Macrophages can inhibit sporangiospore swelling. Growth profiles of non-internalized (), early internalized () and late internalized [before () and after () internalization] *M. circinelloides* sporangiospores (B). Data are represented as median ± IQR of 50 individual sporangiospore measurements (major and minor axes) acquired over time from 3 biologically independent replicates. Statistical significance was assessed by Kruskal–Wallis test followed by Dunn’s multiple comparison test or Mann–Whitney test, as appropriate. Rate of sporangiospore swelling (slope of diameter–time graph) (C). Data are represented as box and whiskers [IQR (boxes), 10–90 percentile (whiskers), median (horizontal line) and outliers (dots)] of 50 individual sporangiospore measurements (major and minor axes) acquired from 3 biologically independent replicates. Statistical significance was assessed by Kruskal–Wallis test followed by Dunn’s multiple comparison test. (D) No correlation was observed between sporangiospore size (major axes measurements) prior to engulfment and speed of sporangiospore swelling inside phagocyte. (E) Germination of resting and pre-swollen sporangiospores was inhibited within the phagocyte. Early internalization = internalized in first 30 min of image acquisition; late internalization = internalized between 210 and 270 min of image acquisition; ∗P≤0.05, ∗∗P ≤0.01, ∗∗∗P ≤0.001, ns = P>0.05.

Our first objective was to establish the impact of the phagosomal environment on the swelling of *A. fumigatus* and *M. circinelloides* spores using live 4D imaging. At start of image acquisition, internalized and non-internalized *A. fumigatus* conidia had similar diameters [median (IQR) = 2.7 μm (2.6–2.8 μm) and 2.7 μm (2.5–2.9 μm), respectively]. By 240 min of image acquisition (before initiation of polarized growth), the diameter of early internalized conidia [median (IQR) = 3.0 μm (2.9–3.2 μm)] was significantly different from non-internalized conidia [median (IQR) = 4.5 μm (4.3–4.8 μm)] conidia (P≤ 0.001). This represented an average 11.0% (6.5–15.8%) increase in diameter size for early internalized conidia, compared to an average 67.1% (58.5 79.7%) increase in diameter size for non-internalized conidia (Fig. 1 A and B). The percentage increase in diameter size for early internalized conidia was significantly different from zero (P≤ 0.001). Similar behaviour was observed for *M. circinelloides*. At start of image acquisition, internalized and non-internalized sporangiospores had similar diameters [median (IQR) = 8.8 μm (7.2–10.9 μm) and 9.8 μm (7.9–11.1 μm)]. By 240 min of image acquisition (before initiation of polarized growth), the diameter of early internalized sporangiospores [median (IQR) = 9.1 μm (7.2 10.7 μm)] was significantly different from non-internalized sporangiospores [median (IQR) = 14.3 μm (11.6 17.2 μm)] (P≤ 0.001). This represented an average 0.7% (-2.5 4.7%) increase in diameter size for early internalized sporangiospores, compared to an average 47.0% (39.1 56.6%) increase in diameter size for non-internalized sporangiospores (Fig. 2 A and B).

These results demonstrate that the phagosome environment alters the dynamics of swelling of spores. Growth of early internalized *A. fumigatus* conidia was delayed, but not impaired, inside a phagocyte. Swelling of early internalized *M. circinelloides* sporangiospores was completely inhibited.

### 3.2 Growth of pre-swollen spores of *A. fumigatus* and *M. circinelloides* is restricted within the phagosome

To evaluate the capacity of macrophages to control growth once swelling had been initiated, late internalized spores were analysed. As expected, prior to uptake no differences in diameter sizes were observed between late internalized and non-internalized spores of *A. fumigatus* [median (IQR) = 3.2 μm (3.0 3.4 μm) and 3.3 μm (3.1 3.4 μm), respectively] or *M. circinelloides* [median (IQR) = 13.2 μm (11.8 14.8 μm) and 12.7 μm (10.5 15.9 μm), respectively]. From the initiation of imaging to the moment prior to uptake, this represented an average increase in diameter of 28.4% (20.4 35.2%) and 41.2% (29.4 51.5%) for late internalized spores of *A. fumigatus* and *M. circinelloides*, respectively. However, from the moment after uptake to end of the experiment, the average increase in diameter of late internalized spores of *A. fumigatus* and *M. circinelloides* decreased to 15.2% (5.5–28.3%) and 0.06% (0.9–2.3%), respectively (Fig. 1 B and Fig. 2 B). These results showed that the phagosomal environment restricts the dynamics of spore swelling even after this process had been initiated.

To compare the capacity of macrophages to control the growth of resting and pre-swollen spores, rates of spore swelling were determined for individual cells in early internalized, late internalized and non-internalized groups. Before engulfment, the rates of swelling for late internalized spores of *A. fumigatus* [mean (IQR) = 0.0048 μm/min (0.0040–0.0059 μm/min)] and *M. circinelloides* [mean (IQR) = 0.018 μm/min (0.015–0.022 μm/min)] were comparable to the rates of swelling of non-internalized spores [mean (IQR) = 0.0049 μm/min (0.0037 0.0059 μm/min) and 0.019 μm/min (0.014 0.024 μm/min), respectively]. After engulfment, the rates of swelling of late internalized spores of *A. fumigatus* [mean (IQR) = 0.0020 μm/min (0.0011–0.0034 μm/min)] and *M. circinelloides* [mean (IQR) = 0.00042 μm/min (0.00037–0.0011 μm/min)] were comparable to those for early internalized spores [mean (IQR) = 0.0016 μm/min (0.0012 0.0019 μm/min) and 0.00018 μm/min (0.00020 0.00077 μm/min), respectively]. In contrast, the rates of swelling of spores outside (non-internalized and late internalized before uptake) and inside phagocytes (early internalized and late internalized after uptake) were significantly different (P≤ 0.001) (Fig. 1, Fig. 2 C).

At the time of uptake, late internalized spores of *A. fumigatus* and *M. circinelloides* were on average 1.2 and 1.6 times bigger than early internalized spores, respectively. However, there was variation in individual spore sizes within the groups. A correlative analysis was performed to determine if macrophages were less efficient at containing the swelling of the larger spores within the groups. No correlation was observed between spore size at time of uptake and spore swelling rates (Fig. 1, Fig. 2 D).

Collectively, these results demonstrate that macrophages are capable of limiting growth of pre-swollen and resting spores to a similar extent.

### 3.3 Polarized growth of *A. fumigatus* and *M. circinelloides* spores is impaired within macrophages

In line with the results previously presented, the germination of early internalized and late internalized spores was dramatically impaired. During the 8 h of imaging, none of the early internalized spores of *A. fumigatus* or *M. circinelloides,* nor the late internalized spores of *A. fumigatus,* formed emergent germ tubes. Only 2.3% of late internalized *M. circinelloides* spores germinated within a phagocyte. In contrast, 89% of non-internalized *M. circinelloides* spores and all of *A. fumigatus* non-internalized spores initiated hyphal growth (Fig. 1 E and Fig. 2 E).

## 4 Discussion

Airborne resting spores of *A. fumigatus* and *M. circinelloides* are inhaled into the lung which presents an environment that is permissive for their germination. The presence of spores in the lung induces neutrophil and macrophage recruitment (Gilbert et al., 2014; Nicolás et al., 2020). Spore swelling is a prerequisite for polarized invasive growth. Therefore, the ability of a macrophage to inhibit germ tube formation from *A. fumigatus* and *M. circinelloides* spores could be enhanced by delaying spore swelling. The first phagocytes to arrive at the site of infection will mainly encounter resting and swollen spores. Previous research has highlighted the ability of phagocytes to inhibit germination of resting spores. However, their activity against swollen spores has remained considerably less studied, despite its clinical relevance (Gilbert et al., 2014; Gresnigt et al., 2018). Here we showed that the phagosome environment of thioglycollate elicited peritoneal macrophages restricts the isotropic swelling of resting and pre-swollen spores, germination and subsequent polarized growth of germ tubes.

As shown in previous studies, elicited peritoneal macrophages do not show fungicidal activity against *A. fumigatus* or *M. circinelloides* (Schaffner et al., 1983)*.* However, the phagosome actively restrains and retards spore growth perhaps due to nutrient limitation within the phagosome. Glucose deprivation has been suggested as a macrophage defence strategy against other pathogens (Gleeson et al., 2016; Lorenz et al., 2004). It is clear that resting spores need a combination of nutritional signals to efficiently germinate (Ekundayo and Carlile, 1964; Osherov, 2009). There is also evidence from studies with other zygomycetes that continued access to nutrients is necessary for maintenance of spore swelling (Ekundayo and Carlile, 1964).

Swollen *Rhizopus* spores have variously been reported as either being similarly phagocytosed to unswollen resting spores (Voelz et al., 2015), or, in a J774A.1 macrophage cell line, swollen spores were reported to be less readily engulfed than resting spores (Itabangi et al., 2022). Some strains of *Rhizpous microsporus* harbour endosymbiotic bacteria such as *Ralstonia pickettii*, that condition the ambient growth medium in a way that inhibits phagocytosis of both dormant and swollen spores (Itabangi et al., 2022).

Spores may need to attain a certain size threshold before initiating polarised growth (Ekundayo and Carlile, 1964). Previous studies suggest this may not be the case, since the provision of nutrients can promote the early onset polarized growth of poorly swollen spores whilst nutrient depletion can prevent germination of fully swollen spores (Ekundayo and Carlile, 1964; Feofilova et al., 2012). More recently, the breaking of dormancy of spores of ascomycete yeasts has been shown to require reduction of the very high internal viscosity or “glassy state” of the spore cytoplasm due to the high internal concentration of compatible solutes such as trehalose (Plante et al., 2023). The transition from a highly viscous to a more fluid cytoplasm more capable of metabolic activity is regulated by chaperones that enable the solubilisation and refolding of the spore proteome (Plante et al., 2023). These events may also herald the events that lead to the swelling of spores of filamentous fungi as more osmotically active compounds become available. The mechanism(s) by which macrophages restrict spore swelling and germination is not yet known, although the phagolysosome environment imparts a number oxidative, nitrosative, pH and other stresses that are likely to be fungitoxic (Erwig and Gow, 2016).

Our data show that diameter of late internalized spores of *A. fumigatus* and *M. circinelloides* could be equal to or larger than the diameter of non-internalized spores immediately before swelling. Therefore, macrophages were able to control the progression of swelling and the establishment of polarized growth, which may be achieved by multiple mechanisms. It would be useful to generate biomarkers specific to each stage of the germination process to enable the progression of this process to be tracked under a range of conditions and during phagocytosis by a range of phagocytic immune cells, including those with defects in specific elements required for the uptake and killing of fungal spores. Though fungal signalling pathways have been implicated in orchestrating spore germination, such as the RAS signalling pathway (Fortwendel et al., 2004), the process is still not sufficiently well understood to genetically track its different stages. Similarly it has been shown in *M. circinelloides* that a non-canonical RNAi pathway regulates many genes that are induced post-phagocytosis (Nicolás et al., 2020). It would be of interest to understand what transcriptional and post-transcriptional processes are related to, or blocked, upon spore phagocytosis.

## 5 Conclusion

Our data show that sentinel phagocytes are able to retard fungal growth of fungal spores at the very earliest stages of spore germination and after isotropic growth has been initiated. This contributes to our understanding that macrophages can retard fungal development at a number of stages in their growth cycle. Ultimately the outcome of disease is a race between the ability of a fungus to establish a foothold of difficult to kill hyphal cells and the immune system to sufficiently retard filamentous development to enable elements of the innate and adaptive immune system to be requited to potential sites of fungal proliferation. The ability to slow or repress spore swelling and germination may be the earliest stage at which fungal suppression takes place in the series of interactions that ultimately determines the success or failure of immunity.

## CRediT authorship contribution statement

María Fernanda Alonso: Conceptualization, Data curation, Formal analysis, Investigation, Methodology, Validation, Visualization, Writing – original draft, Writing - review & editing. Judith M. Bain: Investigation, Methodology, Writing - review & editing. Lars P. Erwig: Conceptualization, Writing - review & editing. Alistair J.P. Brown: Funding acquisition, Conceptualization, Writing - review & editing. Neil A.R. Gow: Conceptualization, Funding acquisition, Project administration, Resources, Validation, Visualization, Writing - original draft, Writing -review & editing.

## Declaration of competing interest

The authors declare that they have no known competing financial interests or personal relationships that could have appeared to influence the work reported in this paper.
